# Faulty Suppression of Irrelevant Material in Patients with Thought Disorder Linked to Attenuated Frontotemporal Activation

**DOI:** 10.1155/2012/176290

**Published:** 2012-04-19

**Authors:** S. M. Arcuri, M. R. Broome, V. Giampietro, E. Amaro, T. T. J. Kircher, S. C. R. Williams, C. M. Andrew, M. Brammer, R. G. Morris, P. K. McGuire

**Affiliations:** ^1^Department of Psychosis Studies, Institute of Psychiatry, King's College London, London, UK; ^2^NIF, Functional Neuroimaging, Lab/LIM 44, Instituto de Radiologia, University of Sao Paulo, Sao Paulo, Brazil; ^3^Warwick Medical School, University of Warwick, Coventry UK and Psychosis Clinical Academic Group, Coventry, UK; ^4^Department of Neuroimaging, Institute of Psychiatry, King's College London, London, UK; ^5^Marburg University, Marburg, Germany; ^6^Department of Psychology, Institute of Psychiatry, King's College London, London, UK

## Abstract

Formal thought disorder is a feature schizophrenia that manifests as disorganized, incoherent speech, and is associated with a poor clinical outcome. The neurocognitive basis of this symptom is unclear but it is thought to involve an impairment in semantic processing classically described as a loosening of meaningful associations. Using a paradigm derived from the n400 event-related, potential, we examined the extent to which regional activation during semantic processing is altered in schizophrenic patients with formal thought disorder. Ten healthy control and 18 schizophrenic participants (9 with and 9 without formal thought disorder) performed a semantic decision sentence task during an event-related functional magnetic resonance imaging experiment. We employed analysis of variance to estimate the main effects of semantic congruency and groups on activation and specific effects of formal thought disorder were addressed using post-hoc comparisons. We found that the frontotemporal network, normally engaged by a semantic decision task, was underactivated in schizophrenia, particularly in patients with FTD. This network is implicated in the inhibition of automatically primed stimuli and impairment of its function interferes with language processing and contributes to the production of incoherent speech.

## 1. Introduction

Bleuler's work in psychosis continues to be highly influential in furthering understanding of the signs and symptoms of schizophrenia [1]. Nevertheless, one of his primary conceptual contributions in understanding schizophrenia, “disturbance of associations” [2, 3], remains to be explained in terms of underlying neural basis. In turn, Bleuler's ideas were influenced by Jung's word association task [4]. Regardless of the psychological or affective mechanisms that may influence the production of speech, Jungian word association is by nature a semantic association test. Interpretations of the broader meanings of “split mind” and “association”, arising from the psychoanalytical field, are not incompatible with an inbuilt characteristic of this test, which taps into the concept of semantic priming [5], extensively investigated in schizophrenia (see Minzenberg et al. [6] for a review in single-word semantic priming in schizophrenia). However, some studies do not take into account specific symptoms proposed by Bleuler, such as formal thought disorder, as an underlying factor that would interfere with task performance (e.g., [7]), mixing up performance of patients with a range of distinct symptoms. Of note, there is a line of investigation suggesting that formal thought disorder (FTD) is associated with hyperactivation of the semantic network [8–14]. These studies mainly employ semantic tasks based on word-pair semantic priming paradigms. It is important to note that such kind of studies, although provided evidence for altered semantic processing associated with FTD, may fail to tap into the type of communication impairment clinically seen in FTD patients. The latter manifests itself in linguistic units that range from large thoughts, such as whole periods with several sentences embedded, to small utterances, such as words within the same sentence [15], or even a single word (e.g., neologism).

Bleuler also suggested that, within its symptoms, FTD is possibly the closest to the neural substrate of schizophrenia [2]. Biological evidence for a neural basis of FTD has been provided by methods employing functional measures of brain activity. EEG studies employing the n400, an event-related potential (ERP) that is a neural marker of semantic processing [16–18], demonstrated a reduction in n400 amplitudes and increased latency in schizophrenia during a sentence processing task [19–21], consistent with impairment in semantic integration, particularly associated with FTD [22–26].

Functional neuroimaging of schizophrenia suggested that FTD is associated with altered resting activity in the medial temporal cortex [27] and with altered activity (brain metabolism or Blood Oxygen Level Dependent-BOLD-activity) during tasks that engage language processing in the dorsolateral prefrontal, bilateral anterior cingulate, and lateral temporal cortices [28–32]). FTD has also been linked to reductions in grey matter volume in the inferior frontal [33, 34] and the temporo-parietal cortex [35]. Using a model of information processing, MacDonalds III and collaborators demonstrated diminished activity in the middle frontal gyrus in schizophrenic patients with disorganization symptoms (includes FTD) associated with context processing deficits [31]. Collectively, the studies particularly implicate the prefrontal, middle frontal, temporal, and anterior cingulate cortices in the pathophysiology of FTD in schizophrenia.

In summary, FTD is a symptom of schizophrenia that manifests itself as disorganized, incoherent speech. Andreasen [36] has operationalized studying this symptom by creating a scale that decomposes the concept of “loosening of meaningful associations” into measurable items. Thus, this clinical manifestation of schizophrenia, which can be objectively measured, is thought to involve impairment in semantic processing, deficits in executive functioning, and altered brain activity in the left frontal, temporal, and anterior cingulate cortices.

The objective of the present study was to use event-related fMRI to examine the extent to which regional activation during semantic processing is altered in schizophrenic patients with FTD. Methodological issues which were not tackled by some of the functional imaging studies mentioned above (small sample sizes, block design fMRI task and an overt production of speech) were carefully addressed. Particularly, we devised a task likely to engage areas associated with linguistic processes with heavier demands [37] using a paradigm derived from semantic decision procedures used in the ERP studies that originally correlated FTD with n400 abnormalities in schizophrenia. We sought to engage the top down modulation of semantic processing by requiring suppression or inhibitory mechanisms to take place, necessary to process semantic incongruent information. Activation of the left inferior frontal and left middle temporal [38, 39], right anterior cingulate [38], and bilateral precuneus [39] was found in studies that required the generation of semantically incongruent endings to complete a previously primed incomplete sentence stem. Therefore, these areas are activated in tasks requiring suppression of endings (meanings) automatically activated by sentences.

We hypothesized the following:

1. Incongruent sentences would engage left inferior frontal, left middle temporal, right anterior cingulate, and bilateral precuneus more than congruent sentences.
2. FTD patients with schizophrenia would show diminished activation relative to controls and patients without FTD in:
  - areas previously demonstrated to show aberrant activity in this group: left prefrontal (middle and inferior), left temporal, and bilateral anterior cingulate cortex and
  - areas associated with manipulation of incongruent material (i.e., the above and also the precuneus).

## 2. Methods

### 2.1. Subjects

Ten healthy adult volunteers and 18 patients meeting DSM-IV criteria for schizophrenia [40] took part in the study. Acutely psychotic patients with either high or low levels of positive FTD were recruited from the South London and Maudsley NHS Trust. Controls were recruited from the same geographical area through local advertisement.

All subjects were dextral [41], males with National Adult Reading Test Revised [42] IQ ≥ 80, and native speakers of British English. Exclusion criteria for controls were a previous history of a neurological or psychiatric disorder, substance dependence, or a medical disorder that could affect the brain. Exclusion criteria for patient were another DSM-IV axis I diagnosis and age of onset of schizophrenia prior to 18 years of age. Subjects received oral and written information about the procedures and gave written consent to participate, with *£*30 in return for participating. The project was approved by the Research Ethical Committee of the Institute of Psychiatry.

Psychopathology was assessed using the Scale for the Assessment of Positive Symptoms (SAPS) [36] and the Scale for the Assessment of Negative Symptoms (SANS) [43]. Positive FTD was assessed on the basis of the corresponding 8 items in the SAPS (derailment; incoherence; illogicality; circumstantiality; pressure of speech; distractible speech; tangentiality; clanging).

In each patient, the score on each FTD SAPS item (range 0–5) was summed to yield a total FTD score (range 0–40). The FTD score was then used both as a classifier to split the patients sample in two subgroups (as described below) and as a continuous measure (see Figure 2(b)), without distinctions within the patients. Thus, patients who had a FTD score 0–4 were classified as non-FTD and those with scores ranging from 5–40 were classified as FTD. The patients sample was split into two groups on this basis producing a subgroup with (FTD, *n* = 9 and a subgroup without FTD (Non-FTD, *n* = 9). There were no significant differences between these subgroup in SAPS total score, SANS total score, scale for the Assessment of Global Functioning (GAF) [40] score, antipsychotic medication dose (in chlorpromazine equivalent), duration of illness, number of psychiatric admissions, or age at first admission (Table 1). There were also no differences in the score for negative FTD, as defined by the SANS items (poverty of speech, poverty of content of speech, thought blocking, and increased latency of response).

The two patient subgroups and the healthy controls had similar demographic characteristics (Table 2).

### 2.2. Task

#### 2.2.1. Sentence Stems

Eighty sentence stems (i.e., last word missing) were used with the degree of constraint previously defined in a large sample drawn from the same population as the control subjects in the present study [44]. Forty stems had a high semantic constraint (HCt) (cloze probability > 0.94) and 40 low semantic constraint (LCt) (cloze probability < 0.34). For example, “He posted the letter without a….”, is a HCt stem (completed by the word “stamp” by 96% of subjects), while “She couldn't imagine anyone less…” is a LCt stem. 

#### 2.2.2. Target Words

Two types of words were presented as target stimuli, taken from completions that had originally been produced for the stems [44]. These words were either congruous with the sentence stem (i.e., semantically related) or incongruous (semantically unrelated). To avoid phonological priming [45], words with the same initial phoneme as the most frequent congruent completion (best completion) for that stem were excluded.

### 2.3. Experimental Paradigm

#### 2.3.1. fMRI Stimuli

**Figure 1:**
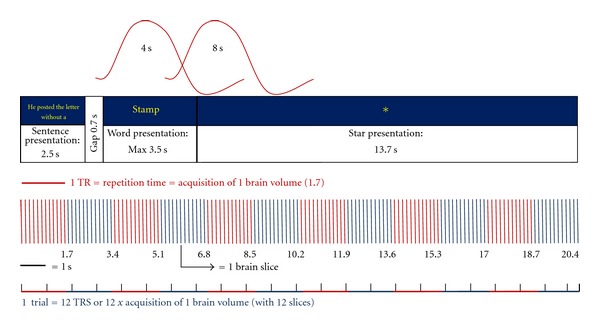
fMRI task design.

Sentence stems were presented onto a screen subtending a visual angle of approximately 1° (text height) by 7°, (stem length). Stems were presented for 2.5 sec. After an interval of 0,7 sec during which the screen was blank, the target word appeared (Figure 1). During the inter stimulus interval, subjects were instructed to fixate on an asterisk for at least 13.7 sec. Eighty trials of 20.4 seconds duration were presented, divided into 5 runs of 16 trials each. Trial type per run was pseudorandomized to control for order effects between subjects. Subjects were instructed to read the sentence stem, then decide if the target completed the stem in a sensible way or not making their choice using one of two buttons on a button box (for accuracy and response time recordings). Prior to scanning, all subjects underwent a training session to make sure they were able to understand and perform the task.

#### 2.3.2. fMRI Data Acquisition

Gradient-echo planar MR images were acquired using a quadrature head coil in a 1.5 Tesla GE Signa System (General Electric, Milwaukee, WS, USA). Head movement was minimised by foam padding and a supporting band across the forehead. A gradient echo EPI axial acquisition (TR 1700 milliseconds-ms, TE 40 ms, FA 90°, matrix 64 × 64, FOV 24 cm, thickness 7 mm, gap 0.7 mm, 192 volumes) was used to collect 12 slices parallel to the intercommissural (AC-PC) plane. The total number of images acquired (in 5 runs) was 960 with just under five minutes between them. Structural images were acquired using gradient echo IR EPI sequence (TR 1600 ms, TE 40 ms, TI 180 ms, matrix 128 × 128, FOV 20 cm, thickness 3 mm, gap 0.3 mm, NEX 8, 43 slices). This latter image was used for normalisation to a standard template.

### 2.4. Analysis

This experiment is part of a larger study [46] in which a series of experiments were designed to test the semantic network and executive functions in FTD patients. The present experiment manipulated semantic constraints and congruencies. In this paper, we only addressed effects of semantic congruency.

### 2.5. Online Behavioral Data

Repeated Measures (SPSS, General Linear Model) analyses were performed for accuracy scores and reaction times (RT) using semantic congruency as within-subject factors. A “yes” response for a semantically incongruent word or “no” response to a congruent word was considered inaccurate. The percentage of correct responses across trials of the same condition was used as within-subject factor.

### 2.6. Neuroimaging Data

Imaging data were analysed with xbam v3.4, Department of Biostatistics and Computing, Institute of Psychiatry, King's College London [47]. More details about the software can be obtained at http://www.brainmap.it/.

#### 2.6.1. Movement Estimation and Correction

The mean signal intensity over all time points (960) was averaged to create a target image (average image intensity at every voxel across all time points) and the sum of absolute differences in grey scale values between the voxels of each observed image (at each time point) and its corresponding base image was computed. A rigid body registration search algorithm was used to estimate the extent of translation and rotation (3 rotations + 3 translations), minimising the total difference between match and base images. The match images were realigned relative to the base image by tricubic spline interpolation and the realigned T2*-weighted time series were regressed on the concomitant and lagged time series of estimated movement at each voxel [48]. Residual time series resulting from the last stage of this procedure were thus uncorrelated with estimated rigid motion in 3D.

#### 2.6.2. Data Analysis

A nonparametric procedure [49] was adopted that avoids assumptions about the distribution of statistics under the null hypothesis. First, the experimental design was convolved with two poisson functions chosen to model the haemodynamic delays of 4 and 8 seconds (see Figure 1). The best fit (weighted sum) of these two convolutions to the observed time series at each voxel was then computed by least squares analysis. The use of the two convolutions within the experimental design allows the time delay between stimulus onset and peak bold response to vary between these limits, which encompass the normal range of haemodynamic delays. Using the parameters of the least squares model fit, the sums of squares of deviations from the mean image intensity over the whole time series due to the model and the residuals were calculated. The ratio of these sums of squares of residuals (model/residuals) was then calculated (and called SSQ ratio). The appropriate null distribution of this statistic was obtained by repeating the model fitting procedure after wavelet-based permutation of the time series [47] twenty times at each voxel and combining the data over all voxels. For any desired type I error rate, the appropriate critical threshold value of SSQ ratio could be obtained from this distribution. Any value of SSQ ratio that exceeded this threshold was deemed to indicate the presence of a voxel responding to the experimental paradigm.

In order to construct group images, the observed and “null” SSQ ratio maps for each experimental condition were then transformed into Talairach space [50]. Median activation maps for each condition were computed after smoothing with a Gaussian filter (FWHM 7.2 mm). Three maps were produced: (1) condition “b” versus “a”, (2) condition “c” versus “a”, and (3) condition “b” versus “c”, where “a” means baseline and “b” and “c” the experimental conditions tested.

The main effect of semantic congruency was assessed by contrasting the 40 congruent trials (20 *low* congruent + 20 *high* congruent) and 40 incongruent trials (20 *low* incongruent + 20 *high *congruent) [conditions “b” and “c” maps]. In order to probe the analysis to the main cognitive process under investigation, we chose the 3rd TR in an event-related design, in which the presentation both of the sentence stem and the final word has already taken place, as shown in Figure 1.

Differences in the responses between conditions were obtained at each voxel by regression of the linear model **F** = **a**_0_ + **a**_1_ **P** + **e**, where the vector of responses in all subjects at a voxel, **P**  is a dummy coding vector expressing the particular contrast between experimental conditions of interest, and **e** is the vector of random errors. Values of **a**_1_* were tested for statistical significance by randomly reallocating the data of each voxel between conditions, thus realising the null distribution under the hypothesis of no difference between responses to different experimental conditions. The analyses reported in this study were carried out using cluster-level statistics to avoid harsh multiple comparison corrections required if each voxel is tested individually [51]. Initially, maps of **a**_1_* were thresholded retaining only clusters with a probability of type I error = 0.0001, for main effects of the task in healthy controls. However, the patients group showed a diminished overall strength of activation relative to healthy controls. We thus decided to report results from maps thresholded at a voxel/cluster probability of type I error = 0.05/0.01, adjusted to get less than 1 false positive per map, to facilitate interpretation of the results, particularly the differences found between controls and patients and between patients themselves (FTD and Non-FTD). The integral of the SSQ ratio values for each of the resulting three-dimensional clusters was then tested against the distribution of cluster SSQ ratio integrals generated after random reallocation of data between conditions at each voxel (see above). Maps reflecting the contrast between conditions (i.e., congruent versus incongruent) in each group were analysed with ANOVAS, producing 2 maps, one showing regions more activated in the first condition and the other showing the reverse.

To minimize the potentially confounding effect of differences in task performance on brain activation, all incorrect trials were excluded from the image analysis. Thus the neuroimaging data were derived from trials in which subjects correctly decided whether the final word was or not semantically congruent with the previous sentence stem.

#### 2.6.3. Data Analyses to Compare Groups

**Figure 2:**
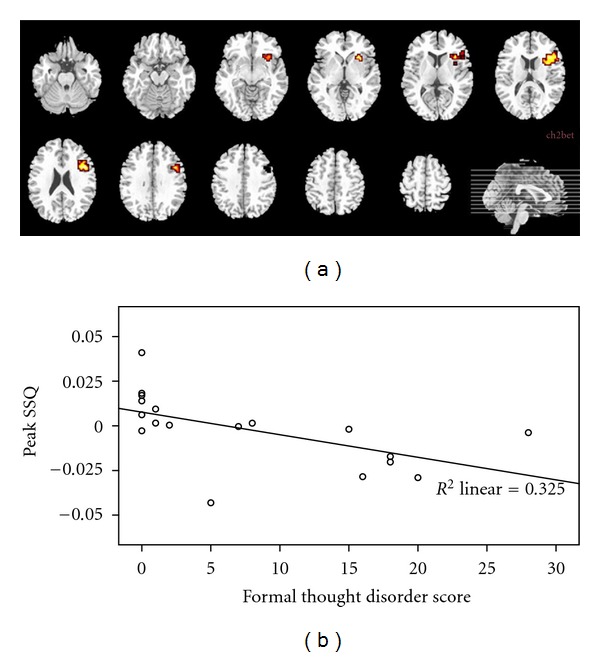
(a) 3-Group ANOVA: semantic congruency (congruent versus incongruent) trials. Comparison of healthy controls (*n* = 10) versus schizophrenic patients with formal thought disorder (FTD, *n* = 9) versus schizophrenic patients without formal thought disorder (Non-FTD, *n* = 9). Attenuated activation in the left inferior frontal gyrus was observed in FTD relative to Non-FTD and to controls. The latter showed greater activation in this region also relative to non-FTD. The left side of the brain images corresponds to the right side of the brain. The superior part of the brain images corresponds to the anterior brain region. (b) Scatter plot of the activity in the left inferior frontal cortex as a function of the score in formal thought disorder (FTD) within schizophrenic patients (*n* = 18). We observe that FTD higher scores in a sample of psychotic schizophrenic patients are negatively correlated with activity in the left inferior frontal cortex (*x* = −43.33, *y* = 14.81, *z* = 14.85).

To investigate between group differences due to effects of semantic congruency, the images resulting from the contrast between semantic congruent versus incongruent trials were directly compared between groups, first contrasting all groups in a 3-Group ANOVA with cluster probability of type I error set at 0.01. This analysis produced a range of maps in which one group showed greater activation than the other groups and vice-versa. These maps are presented in the results section below (Figure 2 and Table 3).

Further analyses were conducted to investigate the nature of the results arising from the semantic congruency analysis: we wanted to determine whether differences in activation maps observed between controls and patients could be due to a specific pattern of activation associated with FTD or not. Therefore, separate post-hoc ANOVAS contrasting FTD with controls and FTD with Non-FTD were performed.

## 3. Results

### 3.1. Online Behavioral Responses

#### 3.1.1. Accuracy

We did not observe an effect of semantic congruency in performance accuracy (*F* = 0.65, *P* = 0.43, *df* = 1). Equally, there was no significant interaction between congruency and group (*F* = 0.46, *P* = 0.64, *df* = 1). Post- hoc tests (Tukey) demonstrated that there was a significant contrast between FTD and controls [mean Difference = −0.128; Std error = 0.397; *P* = 0.009; CI: −0.227 to −0.029], reflecting impairment seen in incongruent trials (Figure 4) with FTD showing the worse performance (more mistakes than both other groups). Non-FTD showed an intermediate level of performance, making more mistakes than FTD and fewer relative to controls, without significant differences in both contrasts.

#### 3.1.2. Reaction Times

In all groups, responses to congruent trials were faster than incongruent ones (see Figure 5), with an observed significant effect of semantic congruency on reaction times (*F* = 14.17, *P* = 0.001, *df* = 1) and no significant interactions between semantic congruency and group (*F* = 0.84, *P* = 0.442, *df* = 2). A robust main effect of group on RT was found (*F* = 34.10, *P* < 0.001, *df* = 2). Non-FTD patients were slower than FTD and controls in both conditions (congruent and incongruent trials). Controls were faster than FTD in both conditions. Post-hoc contrasts demonstrated significant differences in RT between all pairs contrasted, that is, controls versus FTD [mean difference = −0.442; std error = 0.800; *P* < 0.001; CI: −0.639 to −0.244], controls versus NON-FTD [mean difference = −0.672; std error = 0.085; *P* < 0.001; CI: −0.880 to −0.463], and FTD versus NON-FTD [mean difference = −0.229; std error = 0.088; *P* = 0.036; CI: −0.446 to −0.013].

## 4. Neuroimaging Results

### 4.1. Control Subjects

#### 4.1.1. Congruent and Incongruent Sentences (Table 3)

Congruent trials were associated with increased activation relative to incongruent trials in the dorsal portion of the left inferior frontal gyrus, the orbital portion of the right inferior frontal gyrus, the anterior cingulate bilaterally, the right caudate nucleus, the left posterior middle temporal gyrus, the left parahippocampal gyrus, the right precuneus, and the right inferior frontal gyrus. Conversely, incongruent trials were associated with more activation than congruent trials in the left middle frontal gyrus, the right medial frontal gyrus, and the left superior frontal gyrus.

#### 4.1.2. Between Group Differences: Controls versus Non-FTD versus FTD (Table 3)

The contrast between congruent and incongruent sentences was associated with greater activation in the dorsal portion of the left inferior frontal cortex in controls than Non-FTD patients, which in turn had increased activation relative to FTD patients in the same region (Figure 2(a)). In this analysis, we did not find regions in which the patients groups showed more activation than controls.

#### 4.1.3. Activation Associated with FTD

**Figure 3:**
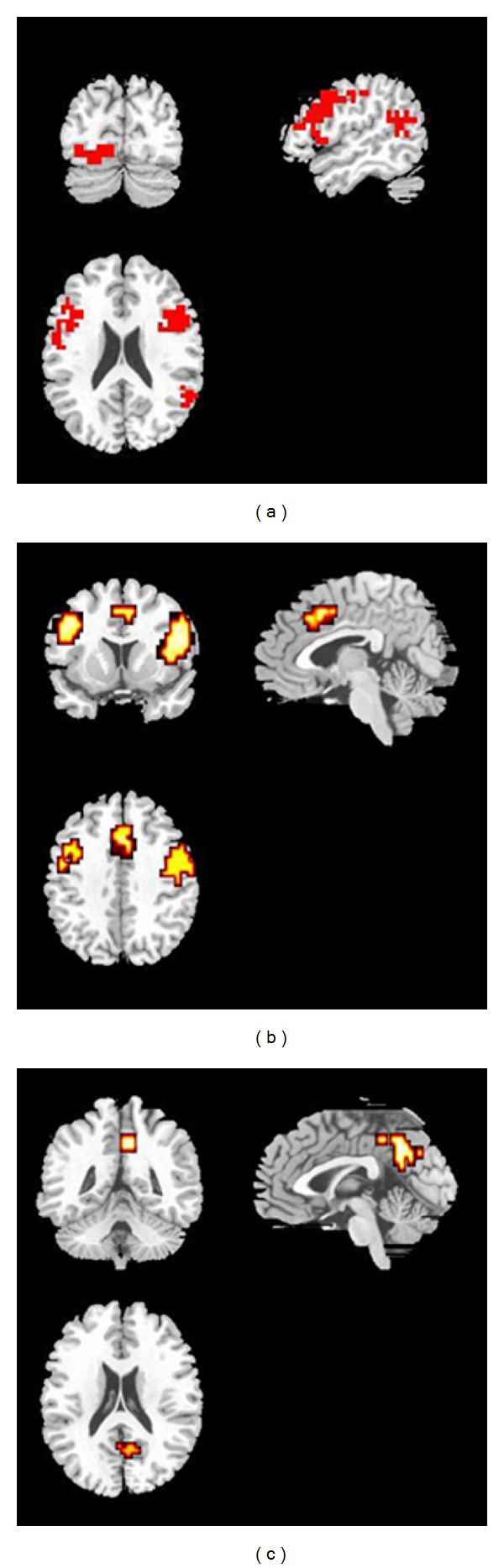
Post-hoc 2 group comparisons: semantic congruency (congruent versus incongruent trials). (a) healthy controls (*n* = 10) versus schizophrenic patients with formal thought disorder (FTD, *n* = 9) (b, c) FTD versus schizophrenic patients without formal Thought Disorder (Non-FTD, *n* = 9). (a) Controls show greater activation relative to FTD in the inferior frontal gyrus bilaterally, dorsal portions, in the left middle temporal gyrus (BA 22), in the left lingual gyrus, in the left precuneus, and in the cerebellum (posterior lobe) bilaterally. (b) Non-FTD patients showed greater activation of bilateral middle frontal gyrus and bilateral anterior cingulate, relative to FTD. (c) FTD patients show greater activation relative to Non-FTD patients in the right posterior cingulate. The left side of the brain images correspond to the right side of the brain. The superior part of the brain images correspond to the anterior brain region.

**Figure 4:**
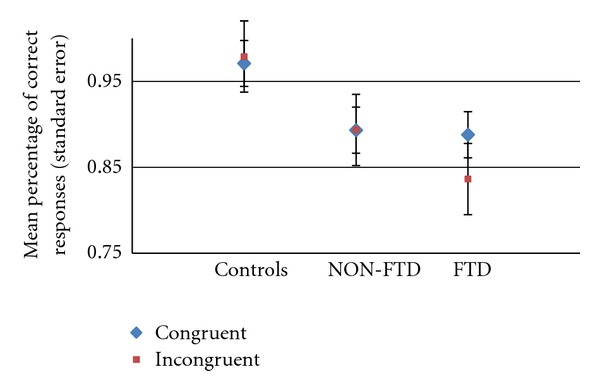
Semantic decision match fMRI task: performance accuracy in semantic congruent and incongruenet trials. Control (*n* = 10) versus NON-FTD (*n* = 9) versus FTD (*n* = 9).

POST-HOC contrasts of FTD patients versus controls and FTD versus Non-FTD patients confirmed attenuated activation in the left inferior frontal cortex in the FTD group. Relative to controls, FTD patients showed less activation in the dorsal portion of the inferior frontal cortex bilaterally, the left middle temporal gyrus, precuneus, and lingual gyrus and in the cerebellum bilaterally (Figure 3(a)). No areas were relatively more activated in the FTD than the control group.

Relative to Non-FTD patients, FTD patients showed less activation in the left middle frontal gyrus and bilaterally in the anterior cingulate gyrus (Figure 3(b) and Table 3). Conversely, the FTD subgroup showed more activation than the Non-FTD subgroup in the right posterior cingulate gyrus and the left cerebellum (Figure 3(c)).

## 5. Discussion

### 5.1. Behavioral Results

**Figure 5:**
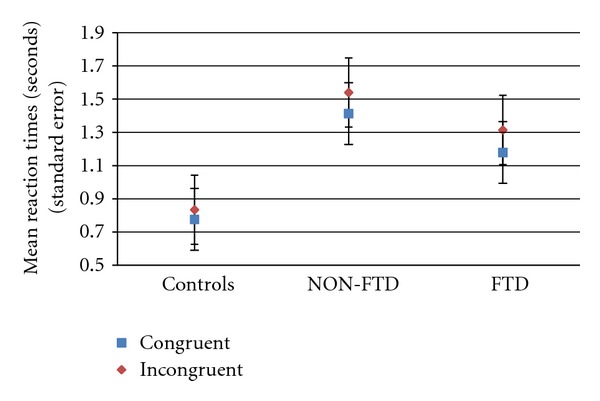
Semantic decision match fMRI task: reaction times in semantic congruent and incongruent trials. Control (*n* = 10) versus NON-FTD (*n* = 9) versus FTD (*n* = 9).

Semantic congruency influenced RTs, with minimum values in trials with congruous endings, as expected (see Figure 5). Therefore, we observed an effect of inhibition (or interference) associated with processing of incongruent sentences in all groups. The patients made more errors than controls, particularly those with FTD. This was most evident on incongruent trials where patients with FTD performed much worse than both patients with no FTD and controls, showing deficiency in processes where inhibition is expected.

### 5.2. Neuroimaging Results

#### 5.2.1. Semantic Processing in Healthy Controls

We found that incongruent sentences activated the left middle frontal cortex more than congruent ones, an area associated with suppression when the same type of semantic material was employed [39] and with inhibition of stereotyped responses [52]. However, in our study, we observed greater activity for *congruent* than incongruent trials in healthy controls in the right precuneus, contrary to our hypothesis. Therefore, our first hypothesis was partially confirmed. A possible explanation for these differences observed between ours and Allen et al.'s study may be the fact that their task required overt production of a final completing word for visually presented priming sentence stems, whilst ours only required making a decision upon a visually presented final word.

The general pattern of activation we found was expected because the semantic task used placed high demands on executive control, such as maintaining competing items on hold whilst checking for semantic appropriateness [53] and inhibiting unnecessary items. Thus, in healthy controls, we observed more activation for congruent than incongruent trials in the right inferior (orbital) and left inferior (dorsal) cortices, left posterior temporal cortex, bilateral anterior cingulate, and left precuneus. Our findings are in line with data supporting an important role of the left hemisphere in the final integration of semantic aspects present in sentences and texts [54] and recent meta-analytical studies showing activity in these regions associated with semantic processing [55–57].

#### 5.2.2. Semantic Processing in FTD Schizophrenic Patients

We also hypothesized that FTD patients would show decreased activation in the prefrontal (middle and inferior), temporal, and cingulate cortices. Relative to controls, FTD patients showed a diminished activation in dorsal inferior frontal cortex (bilaterally) and in the left middle temporal gyrus when processing incongruent relative to congruent sentences. We predicted that these differences would also be evident when contrasting patients with and without FTD. We found that for the incongruent/congruent contrast, the FTD subgroup showed less activation than Non-FTD patients bilaterally in the left inferior/middle frontal gyri and the anterior cingulate gyri. Thus, our second hypothesis was confirmed. The differential brain activity observed in FTD patients while performing a task, without mistakes, seem to be arising from an early point of the semantic processing, since these attenuated activations were observed in areas in which controls showed increased activation for congruent trials relative to incongruent ones and *not* the other way round (contrary to our initial prediction). Although this may seem a contradictory finding, the current study confirms altered brain activity in these areas specifically associated with FTD in schizophrenia [28–32].

Manipulation of incongruent sentences requires overcoming a prepotent response, that is, suppression of automatically activated word(s) after a primed sentence stem [36]. The latter process needs to be maintained “active” for a longer period. Activity in the DLPFC has been associated with working memory and contextual information in experiments requiring heavier semantic processing demands [31, 58]. These group differences in inferior frontal and left middle temporal responses reflected less activation of these areas in patients with FTD relative to both controls and patients without FTD. This differential pattern of response is similar to that evident in their behaviour, where patients with FTD performed markedly worse than both other groups on trials involving semantically incongruent sentences, making more mistakes and also having a shorter RT than Non-FTD patients. Relative to Non-FTD, FTD also had less activation in the anterior cingulate cortices, which have been suggested by several authors to participate in demanding processes, such as monitoring conflict of response [59], engagement of cognitive control [60], top-down inhibitory modulation [61], detection of mnemonic competition, and retrieval induced forgetting [62].

A final point should be made about the activity in the precuneus. This region has been demonstrated to be involved in semantic processing [39, 57, 63, 64] episodic memory retrieval [65], retrieval-induced facilitation [66], and dopamine regulation of working memory [67]. FTD patients, but not Non-FTD patients, had less activation in the left precuneus than controls. Despite some subtle inconsistencies in hemispheric localization (not rare in fMRI studies of schizophrenia), this finding suggests a problem in episodic memory retrieval that might have contributed to the FTD group worse task performance. Thus, FTD patients showed robust diminished activation in areas implicated in the suppression of irrelevant material as well as detection and resolution of mnemonic conflict [68], selecting context-appropriate meanings in the presence of competing meanings [69], and differences between impaired and facilitated information [66]. Interestingly, Arcuri [46] found that FTD patients produced significantly more expected words than controls, when overtly producing anomalous endings for sentences of a high degree of constraint, in a modified version of the Hayling Task [70]. That study included the scanned FTD patients (*n* = 9) from the present study but had a larger sample of FTD patients (*n* = 21). Thus, FTD patients who showed diminished activation in areas implicated in aspects of executive control, when deciding whether a final target word is semantically appropriate for a previously presented sentence, also had difficulties in inhibiting automatically activated words that followed sentences of high semantic constraint, when they were asked to openly generate semantically incongruent completions for those sentences.

The differences between FTD and the Non-FTD patients are not attributable to sociodemographic or other clinical differences as they are matched in all respects other than the severity of positive FTD. Moreover, although the FTD patients performed the task more poorly than Non-FTD patients and controls, the image analysis was restricted to correct trials, indicating that the differences in activation were not simply secondary to the more impaired task performance in this subgroup. The differences in regional responses may thus be linked to the pathophysiology of incoherent speech. The prominent role of the inferior frontal cortex in language processing and particularly semantic processing is consistent with this suggestion. However, the fact that a qualitatively similar but less severe reduction in activation was evident in patients with no FTD indicates that findings in this area are also associated with the disorder of schizophrenia, independent of the presence of this particular symptom.

Liddle et al. [27] proposed that the disorganisation syndrome (disorders of the form of thought and innappropriate affect) is associated with a differential pattern of brain activity in frontal, temporal, and cingulate cortices relative to the two other syndromes (psychomotor poverty and reality distortion). In our sample, although we focused specifically on the symptom of FTD, it was significantly correlated with inappropriate affect, that is, patients with FTD had higher scores in innappropriate effect as well, pointing to the same direction of a distinct syndrome of disorganisation within schizophrenia. 

### 5.3. Limitations

The sample was small, and larger studies should be done to confirm our results and possibly explain the limitations in the observed data, which might have contributed to a possible lack of power in our analyses.

## 6. Conclusion

The frontotemporal network normally engaged by a semantic decision task was underactivated in schizophrenia, particularly in patients with FTD. This network is implicated in the inhibition of automatically primed stimuli and impairment of its function interferes with language processing and contributes to the production of incoherent speech.

## Figures and Tables

**Table 1:** Clinical characteristics of the Patient Groups.

Group	Non-FTD	FTD	*P* value
Positive FTD	0 (1) 0–2	17 (11) 5–20	<**0.001^**a**^**
Negative FTD	5 (7) 0–9	6 (6) 3–14	0.546^a^
SAPS (sum)	23 (16) 8–50	14 (10) 6–26	0.471^b^
SANS (sum)	19 (9) 1–34	27 (13) 11–52	0.588^b^
GAF	31 (13) 21–51	30 (14) 20–40	0.546^a^
Chlorpromazine equivalent in (mg/day)	700 (967) 133–1800	517 (550) 320–1200	0.772^a^
Duration of illness (in years)	6 (12) 0.5–17	11 (14) 8–28	0.190^a^
Hospital admissions			
Number	2 (4) 1–15	6 (9) 1–20	0.142^a^
Age at 1st	25 (9) 19–60	26 (11) 15–31	0.222^a^

^a^Mann-Whitney [median (interquartile range) min and max], ^b^ANOVA [Mean (SD) and range], ^c^Fischer Exact.

**Table 2:** Demographic Description of the Sample.

Groups	AGE Median (interquartile range); min and max	Nart IQ Mean (SD); range	Years of full-time education median (interquartile range); min and max
Controls	35 (10); 24–54	108.3 (13.5); 89–126	14.3–(5.3); 9–24
NON-TD	38 (21); 24–63	101.4 (12.7); 86–122	11.0 (1.5)*; 9–15
TD	33 (16); 23–55	99.2 (10.7); 82–115	11.0 (3)*; 9–12
Analysis results	*P* = 0.222^a^	*P* = 0.267^b^	*P* = 0.029^a^

^a^Kruskal Wallis, ^b^one-way ANOVA, *no difference between TD and non-TD patients for years of education.

**Table:** (a) Within group analysis: congruent versus incongruent trials in healthy controls (*n* = 10). Displayed only clusters > or = 6 in size

Contrast	Size	Tal(*x*)	Tal(*y*)	Tal(*z*)	Probability	Cerebral region
Congruent > Incongruent	2189	43.33	14.81	14.85	0.000656	Left inferior frontal gyrus, dorsal portion
	312	46.94	29.63	−7.15	0.000656	Right inferior frontal gyrus, orbital portion
	181	0.00	11.11	31.35	0.000656	Bilateral anterior cingulate gyrus
	3	14.44	11.11	25.85	0.008530	Right anterior cingulate gyrus
	15	10.83	22.22	−1.65	0.000656	Right caudate
	7	−36.11	−48.15	3.85	0.001969	Left middle temporal gyrus
	6	−21.67	−29.63	−12.65	0.001969	Left parahippocampal gyrus
	6	21.67	−66.67	36.85	0.004593	Right precuneus

Incongruent > Congruent	3094	−32.50	37.04	25.85	0.000678	Left middle frontal gyrus
	15	3.61	44.44	25.85	0.004749	Right medial frontal gyrus
	14	−21.67	55.56	9.35	0.000678	Left superior frontal gyrus

**Table:** (b) Between Groups Analyses:. 3-Group ANOVA: Semantic Congruency (congruent versus incongruent) trials. Comparison of healthy controls (*n* = 10) versus schizophrenic patients with Formal Thought Disorder (FTD, *n* = 9) versus schizophrenic patients without formal thought disorder (Non-FTD, *n* = 9)

Results	Size	Tal(*x*)	Tal(*y*)	Tal(*z*)	Probability	Cerebral Region
Controls > NTD > TD	79	−43.33	14.81	14.85	0.007784	Left inferior frontal gyrus, dorsal portion

**Table:** (c) Post-hoc 2 group comparisons: semantic congruency (congruent versus incongruent trials) schizophrenic patients with formal thought disorder (FTD, *n* = 9) versus healthy controls (*n* = 10) and FTD versus schizophrenic patients without formal thought disorder (Non-FTD, *n* = 9)

Results	Size	Tal(*x*)	Tal(*y*)	Tal(*z*)	Probability	Cerebral Region
Controls > TD	172	−43.33	14.81	14.85	0.000284	Left inferior frontal gyrus, dorsal portion
	95	28.89	−70.37	−18.15	0.000851	Right cerebellum, posterior lobe, declive
	92	50.56	25.93	14.85	0.002553	Right inferior frontal gyrus, dorsal portion
	67	−57.78	−37.04	3.85	0.011211	Left middle temporal gyrus
	42	−25.28	−62.96	36.85	0.012766	Left Precuneus
	35	−39.72	−62.96	−18.15	0.012482	Left cerebellum, posterior lobe, declive
	25	−10.83	−92.59	−7.15	0.012482	Left lingual gyrus

NON-FTD > TD	185	−43.33	14.81	20.35	0.000256	Left middle frontal gyrus
	127	46.94	18.52	25.85	0.001282	Right middle frontal gyrus
	37	0.00	18.52	36.85	0.008720	Bilateral anterior cingulate
NON-FTD < TD	81	7.22	−55.56	20.35	0.003912	Right posterior cingulate
